# Ribonucleoprotein Assembly Defects Correlate with Spinal Muscular Atrophy Severity and Preferentially Affect a Subset of Spliceosomal snRNPs

**DOI:** 10.1371/journal.pone.0000921

**Published:** 2007-09-26

**Authors:** Francesca Gabanella, Matthew E. R. Butchbach, Luciano Saieva, Claudia Carissimi, Arthur H. M. Burghes, Livio Pellizzoni

**Affiliations:** 1 Dulbecco Telethon Institute, Institute of Cell Biology, Monterotondo Scalo, Rome, Italy; 2 Department of Molecular and Cellular Biochemistry, Ohio State University, Columbus, Ohio, United States of America; 3 Center for Motor Neuron Biology and Disease, Department of Pathology, Columbia University Medical Center, New York, New York, United States of America; Centre de Regulació Genòmica, Spain

## Abstract

Spinal muscular atrophy (SMA) is a motor neuron disease caused by reduced levels of the survival motor neuron (SMN) protein. SMN together with Gemins2-8 and unrip proteins form a macromolecular complex that functions in the assembly of small nuclear ribonucleoproteins (snRNPs) of both the major and the minor splicing pathways. It is not known whether the levels of spliceosomal snRNPs are decreased in SMA. Here we analyzed the consequence of SMN deficiency on snRNP metabolism in the spinal cord of mouse models of SMA with differing phenotypic severities. We demonstrate that the expression of a subset of Gemin proteins and snRNP assembly activity are dramatically reduced in the spinal cord of severe SMA mice. Comparative analysis of different tissues highlights a similar decrease in SMN levels and a strong impairment of snRNP assembly in tissues of severe SMA mice, although the defect appears smaller in kidney than in neural tissue. We further show that the extent of reduction in both Gemin proteins expression and snRNP assembly activity in the spinal cord of SMA mice correlates with disease severity. Remarkably, defective SMN complex function in snRNP assembly causes a significant decrease in the levels of a subset of snRNPs and preferentially affects the accumulation of U11 snRNP—a component of the minor spliceosome—in tissues of severe SMA mice. Thus, impairment of a ubiquitous function of SMN changes the snRNP profile of SMA tissues by unevenly altering the normal proportion of endogenous snRNPs. These findings are consistent with the hypothesis that SMN deficiency affects the splicing machinery and in particular the minor splicing pathway of a rare class of introns in SMA.

## Introduction

Spinal muscular atrophy (SMA) is an autosomal recessive disorder characterized by degeneration of motor neurons in the spinal cord and skeletal muscle atrophy. SMA is the leading genetic cause of death in infancy and is classified into three types based on the age of onset and clinical severity [1]–[5]. Type I is the most severe and frequent form of SMA with disease onset before six months of age and death usually by the age of two. Type II is the intermediate form with onset prior to eighteen months and patients never gaining the ability to walk. Type III is the mild form characterized by onset after eighteen months with the ability to walk and a normal life expectancy. Very severe fetal-onset (type 0) and very mild adult-onset (type IV) forms of SMA have also been described. Irrespective of disease severity, all SMA patients have homozygous deletions or mutations in the survival motor neuron (*SMN1*) gene, the SMA-determining gene, and retain at least one copy of the nearly identical *SMN2* gene [6]. A single nucleotide difference functionally distinguishes *SMN1* from *SMN2*, affecting the amount of exon 7 inclusion in the SMN transcript [7], [8]. The loss of exon 7 removes the carboxy-terminal amino acids making SMNΔ7 protein unstable and rapidly degraded [9]. As a consequence, the *SMN2* gene produces low levels of full-length SMN and cannot compensate for the loss of *SMN1* in SMA. However, the *SMN2* gene copy number acts as a modifier of disease severity [10], which inversely correlates with the levels of SMN expression in patients [11], [12].

The *SMN* gene is ubiquitously expressed and is essential for viability in many organisms from yeast to humans. To understand SMA etiology and support therapeutics development, a number of different animal models of SMA have been created and SMA mice more closely resemble the feature of SMA pathology in humans [3], [13]. Distinct strategies have been employed to circumvent the early embryonic lethality associated with the knockout of the single *Smn* gene in mice and generate mouse models of SMA [14]–[17]. Similar to the situation in the human disease, expression of the human *SMN2* gene in the mouse *Smn* null background rescues embryonic lethality and results in mice with SMA [15], [16]. Importantly, the severity of the phenotype is dependent on the *SMN2* copy number. Severe SMA mice with a single *SMN2* gene die embryonically while mice with two *SMN2* copies survive on average for five days [15], [16]. These mice have reduced weight and severe motor impairment before appreciable loss of motor neuron cell bodies, which occurs at postnatal day 3. Eight or more *SMN2* gene copies fully rescue the SMA phenotype in SMA mice [16]. Interestingly, introducing a SMNΔ7 transgene that encodes a SMN isoform lacking exon 7–which is the predominant isoform produced by the *SMN2* gene–to a severe SMA mouse genetic background has moderately beneficial effects on survival [18]. SMNΔ7 SMA mice still display a considerably severe phenotype and die on average at two weeks of age. A mouse model of the mild form of SMA was generated by introducing SMN(A2G), a SMN point mutant found in type III SMA patients, to the severe SMA background [19]. SMN(A2G) SMA mice display very late onset of muscle weakness and motor impairment, moderate loss of motor neurons, and survive over one year. Collectively, these studies indicate that reduced SMN activity causes SMA and that disease severity inversely correlates with SMN levels in both human patients and mouse models.

The molecular defect responsible for motor neuron degeneration in SMA is unknown. SMN is a multifunctional protein that has been implicated in a variety of cellular processes, many of which are linked to RNA metabolism [20], [21]. It is well established that SMN associates with itself and at least eight additional proteins (Gemins2-8 and unrip) to form a macromolecular complex referred to as the SMN complex [22]. To date, the only molecularly defined cellular activity of the SMN complex is its function in the biogenesis of small nuclear ribonucleoproteins (snRNPs), the essential components of the pre-mRNA splicing machinery that catalyze the excision of introns from mRNA precursors in the nucleus. Spliceosomal snRNPs of the Sm class comprise an snRNA molecule (U1, U2, U4, U4atac, U5, U11 and U12), seven common Sm proteins and additional proteins specific for each snRNP [23]. The snRNP biogenesis pathway requires the activity of a number of auxiliary factors and takes place in both the nucleus and the cytoplasm [22], [23]. In the cytoplasm, the SMN complex mediates the ATP-dependent assembly of a heptameric ring of Sm proteins around a conserved sequence of the snRNAs to form the Sm core [24]–[26]. Consistent with the essential role for the SMN complex in snRNP assembly, reducing expression of either SMN or Gemin proteins in cultured cells affects Sm core formation [27]–[33]. It is, however, becoming increasingly clear that SMN may have additional functions in distinct cell types that are unrelated to snRNP biogenesis and are relevant to SMA [34]–[37]. A subset of SMN complexes that do not contain Sm proteins traffic into axons and growth cones of motor neurons and may be involved in some aspects of the axonal transport and localized translation of specific mRNAs [38], [39]. Accordingly, cultured primary motor neurons from severe SMA mice display reduced accumulation of β-actin mRNA in growth cones and defective axon outgrowth [37].

One of the most pressing issues in SMA biology is whether deficiencies in snRNP biogenesis, axonal SMN function(s) or possibly even both are responsible for motor neuron degeneration in SMA. A link between deficiencies in snRNP biogenesis and SMA pathology is suggested by the observation that some SMN mutants of SMA patients display defective binding to Sm proteins and snRNP assembly activity *in vitro* [31], [40], [41]. Moreover, extracts from human fibroblast and lymphoblast cell lines of type I SMA patients have reduced snRNP assembly activity compared with normal controls [32]. To date, the most direct evidence that defects of snRNP metabolism may trigger motor neuron degeneration in SMA is that injection of snRNPs rescues motor axon outgrowth and pathfinding defects caused by knockdown of SMN in zebrafish embryos [33], [35]. Although in the zebrafish model system is important to distinguish whether motor axon defects are cell autonomous or secondary to general development defects which cause axonal abnormalities [34], [35], knockdown of two additional factors involved in the pathway of snRNP biogenesis reportedly also affects motor axon outgrowth [33]. These findings, together with the observation that SMN activity in snRNP assembly is very prominent during embryonic and early postnatal development but is strongly down regulated after myelination in the mouse spinal cord [42], suggest that developmental deficiencies in snRNP biogenesis may underlie motor neuron degeneration in SMA. According to this view, reduced SMN activity in snRNP assembly would affect the accumulation of snRNPs thereby weakening the splicing machinery. To date, however, there has been no evidence that snRNP levels are affected in SMA. Furthermore, endogenous snRNP levels are not decreased in chicken DT40 cells with markedly reduced SMN levels and impaired Sm core formation as well as in *Drosophila* larvae that harbor lethal SMN null mutations [32], [36]. These observations raise the issue as to whether snRNP assembly activity in SMA ever falls significantly below the threshold that would affect the steady-state snRNP levels *in vivo* [22]. Here, we sought to address the consequences of SMN deficiency on snRNP metabolism in the spinal cord, the target tissue of SMA. To do so, we analyzed SMN complex expression, Sm core formation activity and endogenous snRNP levels in tissues of mouse models of SMA. Our results indicate that disease severity correlates with the degree of impairment of SMN complex expression and snRNP assembly activity in the spinal cord of SMA mice. Remarkably, defective SMN complex function in snRNP assembly causes a significant decrease in the levels of a subset of spliceosomal snRNPs and preferentially affects the accumulation of U11 snRNP of the minor splicing pathway in tissues from severe SMA mice. Thus, SMN deficiency unevenly alters the physiological proportion of endogenous snRNPs in SMA.

## Results

### SMN complex expression in the spinal cord of severe SMA mice

**Figure 1:**
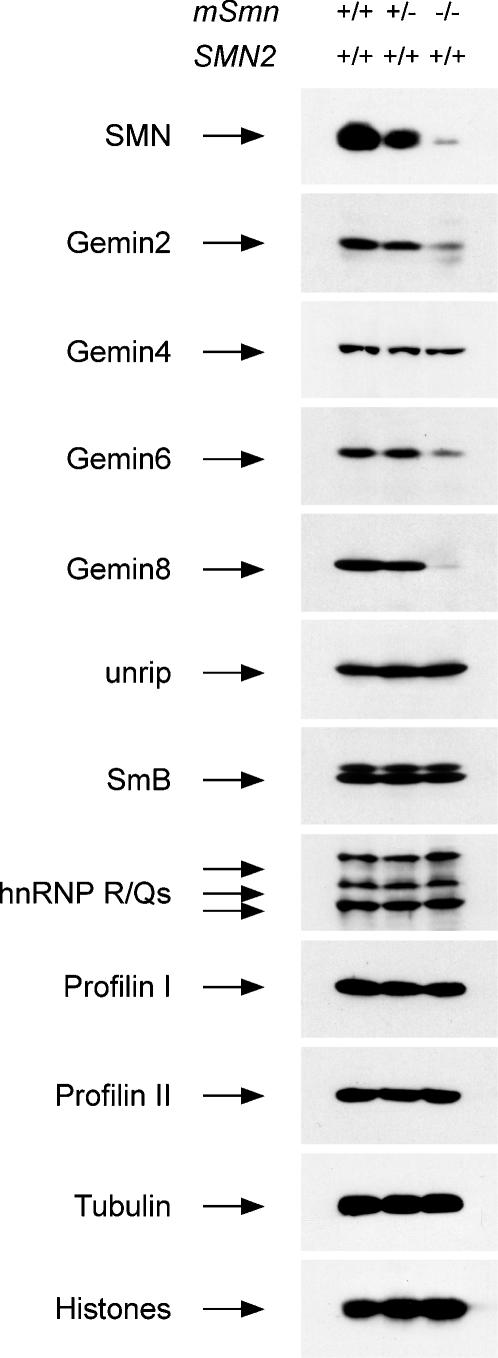
Expression of SMN complex components in the spinal cord of severe SMA mice. Western blot analysis of equal amounts of total proteins from the spinal cord of normal (*SMN2* ^+/+^;*mSmn* ^+/+^), carrier (*SMN2* ^+/+^;*mSmn* ^+/−^) and severe SMA (*SMN2* ^+/+^;*mSmn* ^−/−^) mice at postnatal day 3. All mice harbored two copies of human *SMN2* gene together with two, one or no copies of the mouse *mSmn* gene. Proteins were analyzed by SDS/PAGE and Western blot with antibodies against the proteins indicated on the left.

To analyze the expression of the integral components of the SMN complex in the spinal cord of severe SMA mice, we isolated spinal cords from normal (*SMN2* ^+/+^;*mSmn* ^+/+^), carrier (*SMN2* ^+/+^;*mSmn* ^+/−^) and severe SMA (*SMN2* ^+/+^;*mSmn* ^−/−^) mice at postnatal day 3. Total proteins from these tissues were analyzed by Western blot with antibodies against SMN complex components and several SMN-interacting proteins (Figure 1). Similar levels of tubulin and core histones indicate that equal amounts of proteins were present. Compared with normal controls, the levels of Gemin2, Gemin6 and Gemin8 are strongly decreased in the spinal cord of severe SMA mice that express low levels of SMN. Interestingly, Gemin8 is the SMN complex component whose expression is most affected by reduced levels SMN. In contrast, there is little if any reduction in the expression of Gemin4 and unrip proteins, two other integral components of the SMN complex. Although we did not analyze the levels of Gemin3, Gemin5 and Gemin7 for lack of suitable antibodies, decreasing SMN expression in HeLa cells by RNA interference to an extent similar to that in the spinal cord of SMA mice significantly reduces the levels of Gemin2, Gemin6, Gemin7 and Gemin8 but not of Gemin3, Gemin4, Gemin5 or unrip proteins (Figure S1). Interestingly, the levels of several SMN-interacting proteins such as SmB, hnRNP R, hnRNP Q, profilin I and profilin II are not decreased in the spinal cord of severe SMA mice. Collectively, these results demonstrate that reduced levels of SMN markedly affect the expression of a subset of Gemin proteins and in particular of Gemin8 in the spinal cord of severe SMA mice.

### SMN levels and snRNP assembly defects in tissues of severe SMA mice

**Figure 2:**
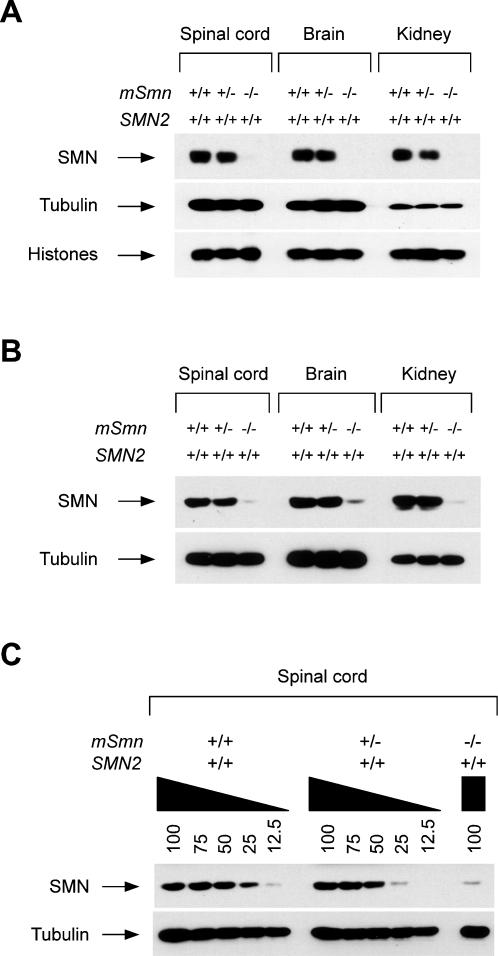
Analysis of SMN levels in different tissues of severe SMA mice. (A) Western blot analysis of total proteins from spinal cord, brain or kidney of normal (*SMN2* ^+/+^;*mSmn* ^+/+^), carrier (*SMN2* ^+/+^;*mSmn* ^+/−^) and severe SMA (*SMN2* ^+/+^;*mSmn* ^−/−^) mice at postnatal day 3. Extracts normalized per cell number (equal amounts of core histones) were analyzed by SDS/PAGE and Western blot with antibodies against the proteins indicated on the left. (B) Western blot analysis of equal amounts of whole tissue extracts (25 µg) from spinal cord, brain or kidney of normal (*SMN2* ^+/+^;*mSmn* ^+/+^), carrier (*SMN2* ^+/+^;*mSmn* ^+/−^) and severe SMA (*SMN2* ^+/+^;*mSmn* ^−/−^) mice at postnatal day 3. Proteins were analyzed by SDS/PAGE and Western blot with antibodies against SMN and tubulin. (C) Analysis of SMN decrease in the spinal cord of severe SMA mice. Western blot analysis of the indicated serial dilutions (100 equals 50 µg of proteins) of whole tissue extracts from the spinal cord of normal (*SMN2* ^+/+^;*mSmn* ^+/+^), carrier (*SMN2* ^+/+^;*mSmn* ^+/−^) and severe SMA (*SMN2* ^+/+^;*mSmn* ^−/−^) mice at postnatal day 3. Proteins were analyzed by SDS/PAGE and Western blot with antibodies against SMN and tubulin.

**Figure 3:**
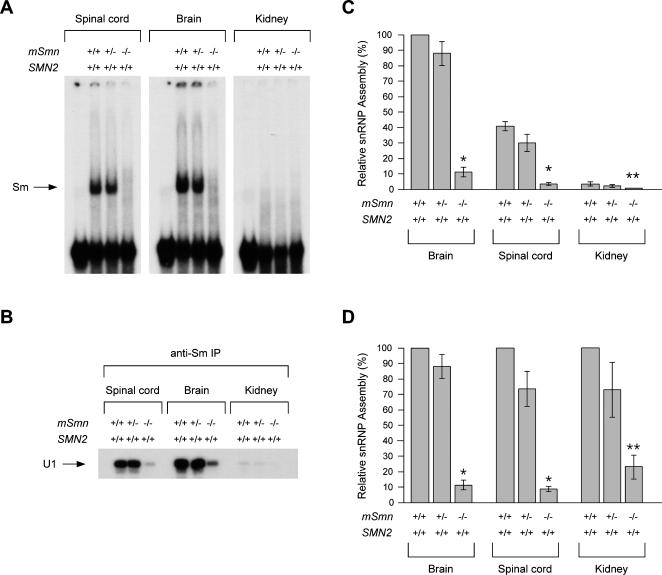
* In vitro* snRNP assembly activity in tissues of severe SMA mice. (A) Equal amounts of whole tissue extracts (25 µg) from spinal cord, brain or kidney of normal (*SMN2* ^+/+^;*mSmn* ^+/+^), carrier (*SMN2* ^+/+^;*mSmn* ^+/−^) and severe SMA (*SMN2* ^+/+^;*mSmn* ^−/−^) mice at postnatal day 3 were analyzed in snRNP assembly reactions with radioactive U1 snRNA followed by electrophoresis on native polyacrylamide gels. The position of U1 RNP complexes containing the Sm core is indicated on the left. (B) *In vitro* snRNP assembly reactions as in (A) were analyzed by immunoprecipitation with anti-Sm (Y12) antibodies followed by electrophoresis on denaturing polyacrylamide gels and autoradiography. (C) Quantification of snRNP assembly activity in whole tissue extracts from normal, carrier and SMA mice. The amount of U1 snRNA immunoprecipitated in snRNP assembly experiments as in (B) was quantified using a STORM 860 Phosphorimager (Molecular Dynamics) and expressed as a percentage of that in brain extracts of normal mice, which is set arbitrarily as 100%. The values from four independent experiments are presented as mean±SEM (*p<0.0001, **p<0.05). (D) For each tissue, the amount of U1 snRNA immunoprecipitated in snRNP assembly experiments with extracts from carrier (SMN2^+/+^;mSmn^+/−^) and SMA (SMN2^+/+^;mSmn^−/−^) mice is expressed as a percentage of that in the corresponding extract from normal (SMN2^+/+^;mSmn^+/+^) mice, which is set arbitrarily as 100%.

The best-characterized function of the SMN complex is in the assembly of the Sm core on spliceosomal snRNAs [22], [23]. To analyze the effect of reduced SMN expression on snRNP assembly in tissues of SMA mice, we isolated spinal cords, brains and kidneys from normal (*SMN2* ^+/+^;*mSmn* ^+/+^), carrier (*SMN2* ^+/+^; *mSmn* ^+/−^) and severe SMA (*SMN2* ^+/+^;*mSmn* ^−/−^) mice at postnatal day 3. For each genotype, Western blot analyses show that these tissues contain similar levels of SMN when normalized per cell number (Figure 2A). This pattern is also observed when equal amounts of whole tissue extracts are compared (Figure 2B). To evaluate the extent of SMN decrease, we compared the intensity of SMN signals detected by western blot in serial dilutions of whole spinal cord extracts from normal, carrier and severe SMA mice. SMN levels in spinal cord extracts from carrier and severe SMA mice are, respectively, two-fold and eight-fold lower than in normal mice (Figure 2C). We then analyzed the capacity of equal amounts of these tissue extracts to form Sm cores on *in vitro* transcribed radioactive U1 snRNA using a well-established ATP-dependent snRNP assembly assay and both gel shift and immunoprecipitation with anti-Sm antibodies [26], [42]. Spinal cord and brain extracts from normal and carrier mice display very efficient snRNP assembly, which is dramatically impaired in the corresponding extracts of severe SMA mice (Figure 3A and 3B). Despite similar amounts of SMN (Figure 2B), kidney extracts from normal and carrier mice show very low Sm core formation compared with extracts from the CNS (Figure 3B) and the reduction in kidney extracts from severe SMA mice is more readily detected upon longer exposure of immunoprecipitation experiments (Figure S2). We have shown previously that snRNP assembly is much more prominent in the CNS than in other mouse tissues during embryogenesis and early postnatal development likely because of changes in the SMN complex activity that occur during development and cellular differentiation [42]. Developmental down-regulation of Sm core formation has been reportedly observed also in *Drosophila* extracts [36]. Thus, differences in the snRNP assembly activity of distinct tissues from normal mice are consistent with these studies. To quantify the snRNP assembly defect in SMA tissues, the levels of immunoprecipitated U1 snRNA were calculated from four independent experiments in which all tissue extracts were prepared and analyzed at the same time as in Figure 3B to minimize variations. Values are presented following normalization either inter-tissue (Figure 3C) or intra-tissue (Figure 3D). The snRNP assembly activity is decreased ten-fold in both spinal cord and brain extracts from severe SMA mice compared with normal mice (p<0.0001) and approximately four-fold in kidney extracts from severe SMA mice compared with normal mice (p = 0.0438). Interestingly, snRNP assembly is only marginally reduced in spinal cord extracts from carrier mice that have approximately half the amount of SMN of normal mice (p>0.05, not significant). Altogether these results show a similar reduction in SMN levels and a strong impairment of snRNP assembly activity in tissues of severe SMA mice, although the defect appears smaller in kidney than in neural tissue.

### snRNP assembly defects in the spinal cord of SMA mice correlate with disease severity

**Figure 4:**
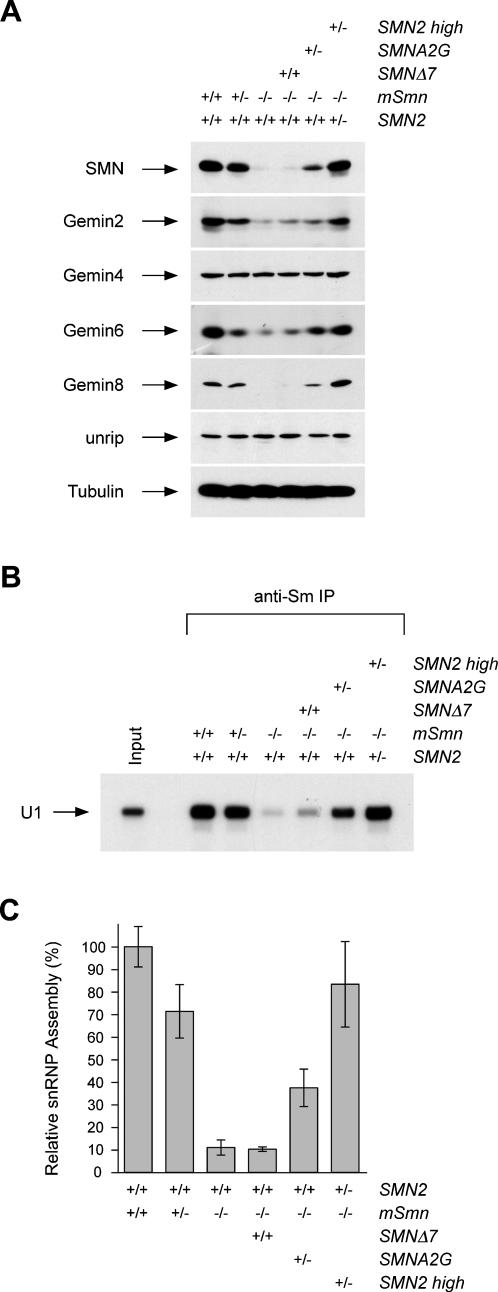
* In vitro* snRNP assembly defects correlate with SMA severity. (A) Western blot analysis of equal amounts of whole tissue extracts (25 µg) from the spinal cord of normal (*SMN2* ^+/+^;*mSmn* ^+/+^), carrier (*SMN2* ^+/+^;*mSmn* ^+/−^), severe SMA (*SMN2* ^+/+^;*mSmn* ^−/−^), SMNΔ7 SMA (*SMN2* ^+/+^ *;SMNΔ7* ^+/+^;*mSmn* ^−/−^), SMN(A2G) SMA (*SMN2* ^+/+^ *;*SMN(A2G)^+/−^ ;*mSmn* ^−/−^) and high copy SMN2 SMA (*SMN2* ^+/−^;*SMN2(566)* ^+/−^;*mSmn* ^−/−^) mice at postnatal day 3. Proteins were analyzed by SDS/PAGE and Western blot with antibodies against the proteins indicated on the left. (B) Representative snRNP assembly reactions carried out using *in vitro* transcribed radioactive U1 snRNA and 25 µg of whole spinal cord extracts as in (A) followed by immunoprecipitation with anti-Sm (Y12) antibodies. Immunoprecipitated U1 snRNAs were analyzed by electrophoresis on denaturing polyacrylamide gels and autoradiography. (C) Quantification of snRNP assembly activity in spinal cord extracts from control and SMA mice of different severity. Spinal cord extracts from at least five mice for each of the indicated genotypes were analyzed by snRNP assembly and immunoprecipitation experiments as in (B). The amount of immunoprecipitated U1 snRNA was quantified using a STORM 860 Phosphorimager (Molecular Dynamics). The values are presented as mean±SEM. *SMN2 high* refers to *SMN2(566)*.

To investigate a potential correlation between impaired snRNP assembly and SMA severity, we analyzed SMN complex expression and snRNP assembly activity in the spinal cord of different mouse models of SMA, including SMNΔ7 SMA mice, mild SMN(A2G) SMA mice and phenotypically rescued high copy SMN2 SMA mice. Figure 4A shows Western blot analysis of SMN complex expression in whole spinal cord extracts from normal (SMN2^+/+^;mSmn^+/+^), carrier (SMN2^+/+^;mSmn^+/−^), severe SMA (SMN2^+/+^;mSmn^−/−^), SMNΔ7 SMA (SMN2^+/+^;SMNΔ7^+/+^; mSmn^−/−^), SMN(A2G) SMA (SMN2^+/+^;SMN(A2G)^+/−^; mSmn^−/−^) and high copy SMN2 SMA (SMN2^+/−^; SMN2(566)^+/−^;mSmn^−/−^) mice at postnatal day 3. The levels of Gemin2, Gemin6 and Gemin8 are strongly decreased in both severe SMA and SMNΔ7 SMA mice compared with normal and carrier mice (Figure 4A). Remarkably, expression of these Gemin proteins is partially restored in the spinal cord of mild SMN(A2G) SMA mice and equivalent to that of controls in the spinal cord of high copy SMN2 SMA mice, which is consistent with the complete rescue of the SMA phenotype. These results highlight a strong correlation between disease severity and decreased expression of Gemin proteins in the spinal cord of SMA mice.

Next, we investigated snRNP assembly activity in spinal cord extracts from the same mice at postnatal day 3. The efficiency of Sm core formation was analyzed by immunoprecipitation of U1 snRNA from snRNP assembly reactions using anti-Sm antibodies and representative results are shown in Figure 4B. Quantification of U1 levels indicates that there is a ten-fold decrease (p<0.0001) in the snRNP assembly activity of spinal cord extracts from both severe SMA and SMNΔ7 SMA mice compared with normal controls (Figure 4C). Importantly, intermediate levels of snRNP assembly activity are found in SMN(A2G) SMA mice, which are approximately three- to four-fold higher than in severe SMA mice (p<0.05) and two-fold lower than in normal controls (p<0.0001). The snRNP assembly activity in spinal cord extracts from normal, carrier and high copy SMN2 SMA mice is not significantly different (p>0.05). This is particularly interesting in the case of carrier mice (*SMN2* ^+/+^;*mSmn* ^+/−^) in which an approximately 50% reduction of SMN levels has very modest if any effects on snRNP assembly activity. Altogether these findings demonstrate that disease severity correlates with the degree of impairment of the SMN complex activity in snRNP assembly in the spinal cord of SMA mice.

### snRNP biogenesis is not affected in human fibroblasts from SMA type I patients

**Figure 5:**
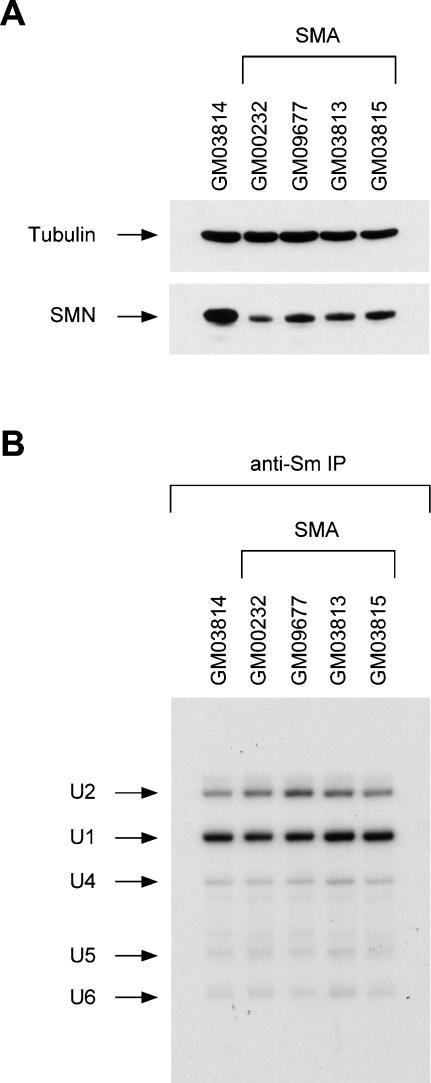
SMN levels and *in vivo* snRNP synthesis in human fibroblasts from type I SMA patients. (A) SMN levels in type I SMA fibroblast cell lines. Western blot analysis of equal amounts of whole cell extracts (25 µg) from human fibroblast cell lines of a normal individual (GM03814) and four type I SMA patients (GM00232, GM09677, GM03813 and GM03815). Proteins were analyzed by SDS/PAGE and Western blot with antibodies against SMN and tubulin. (B) *In vivo* snRNP synthesis in type I SMA fibroblast cell lines. The human fibroblast cell lines in (A) were pulse-labeled *in vivo* for 6 h with [^32^P] phosphoric acid and equal amounts of extracts from these cells (200 µg) were then immunoprecipitated with anti-Sm (Y12) antibodies. The immunoprecipitated snRNAs were analyzed by electrophoresis on denaturing polyacrylamide gels and autoradiography. Known snRNAs are indicated on the left.

Next, we sought to investigate whether reduced SMN levels and snRNP assembly activity resulted in decreased levels of newly assembled snRNPs in SMA cells. To do so, we used well-characterized human fibroblasts cell lines from type I SMA patients. Western blot analysis shows a reduction in SMN levels ranging from 50% to 75% in four different human SMA fibroblast cell lines compared with control fibroblasts from an healthy carrier individual (Figure 5A). Also, snRNP assembly activity in whole extracts from these human SMA fibroblasts is reduced approximately two- to three-fold (data not shown), which is proportional to the decrease in SMN levels and in agreement with the results of a previous study that analyzed Sm core formation in the same cell lines [32]. To study the consequence of this biochemical deficiency on snRNP synthesis *in vivo*, control and type I SMA fibroblasts were grown in the presence of [^32^P] phosphoric acid for six hours to label newly synthesized snRNPs, which were then isolated by immunoprecipitation with anti-Sm antibodies from equal amounts of cell extracts. Surprisingly, we found no significant difference in the levels of snRNPs that are synthesized in control and type I SMA fibroblasts (Figure 5B). Consistent with this, steady-state snRNP levels are also unchanged in these cells (data not shown). Under similar experimental conditions, inihibition of snRNP biogenesis with leptomycin B—a compound that blocks snRNA export from the nucleus [43]—causes a dramatic decrease in the amount of newly synthesized snRNPs indicating that the immunoprecipitation assay is not saturated (data not shown). These results demonstrate that, despite a two- to three-fold reduction in both SMN levels and snRNP assembly activity, snRNP biogenesis is not affected in human type I SMA fibroblasts because fibroblasts contain an excess capacity for Sm core formation relative to their actual requirement for snRNP synthesis *in vivo*.

### The levels of a subset of snRNPs are reduced in tissues of severe SMA mice

**Figure 6:**
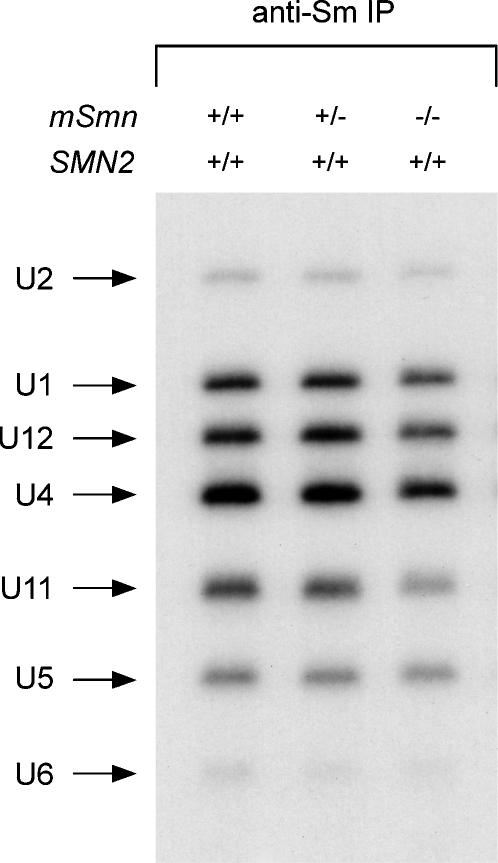
Analysis of endogenous snRNP levels in the spinal cord of severe SMA mice. Equal amounts of whole tissue extracts (200 µg) from the spinal cord of normal (*SMN2* ^+/+^;*mSmn* ^+/+^), carrier (*SMN2* ^+/+^; *mSmn* ^+/−^) and severe SMA (*SMN2* ^+/+^;*mSmn* ^−/−^) mice at postnatal day 3 were immunoprecipitated with anti-Sm (Y12) antibodies. Immunoprecipitated snRNAs were labeled at the 3′-end with [^32^P] pCp and analyzed by electrophoresis on denaturing polyacrylamide gels and autoradiography. Known snRNAs are indicated on the left. Note that the intensity of signal is not proportional to the abundance of individual snRNAs but reflects the efficiency with which they are labeled by T4 RNA ligase [52].

**Figure 7:**
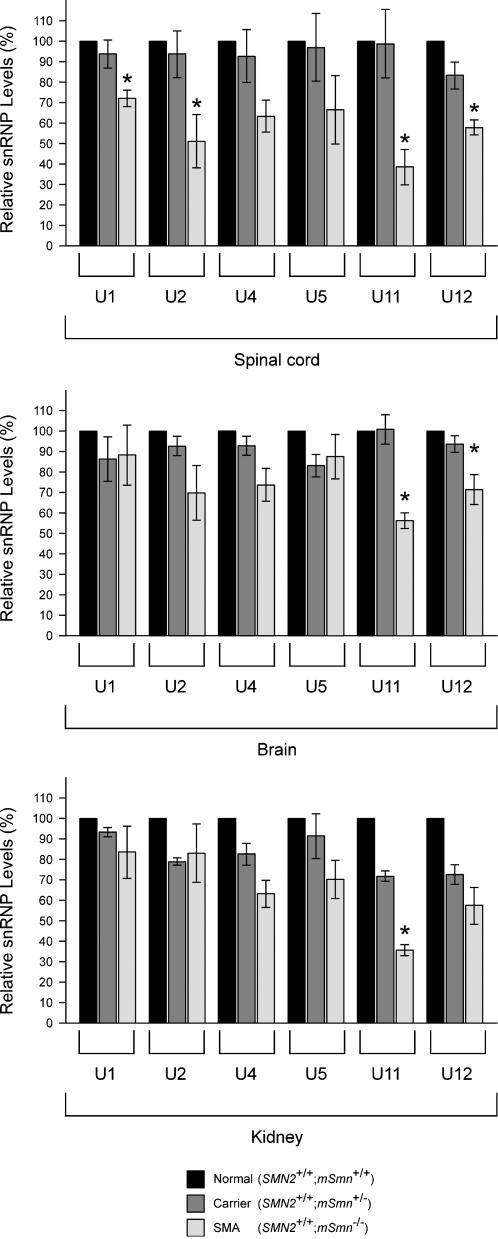
Quantification of endogenous snRNP levels in tissues of severe SMA mice. Immunoprecipitation experiments with anti-Sm (Y12) antibodies from whole tissue extracts (200 µg) from either spinal cord, brain or kidney extracts of normal (*SMN2* ^+/+^;*mSmn* ^+/+^), carrier (*SMN2* ^+/+^;*mSmn* ^+/−^) and severe SMA (*SMN2* ^+/+^;*mSmn* ^−/−^) mice at postnatal day 3 were carried out as in Figure 6. The relative amount of individual snRNAs immunoprecipitated from each tissue was quantified using a STORM 860 Phosphorimager (Molecular Dynamics) and expressed as a percentage of that in normal mice, which is set arbitrarily as 100%. The values from four independent experiments are presented as mean±SEM (*p<0.05). Normal (black bars); carrier (dark grey bars); severe SMA (light grey bars).

**Figure 8:**
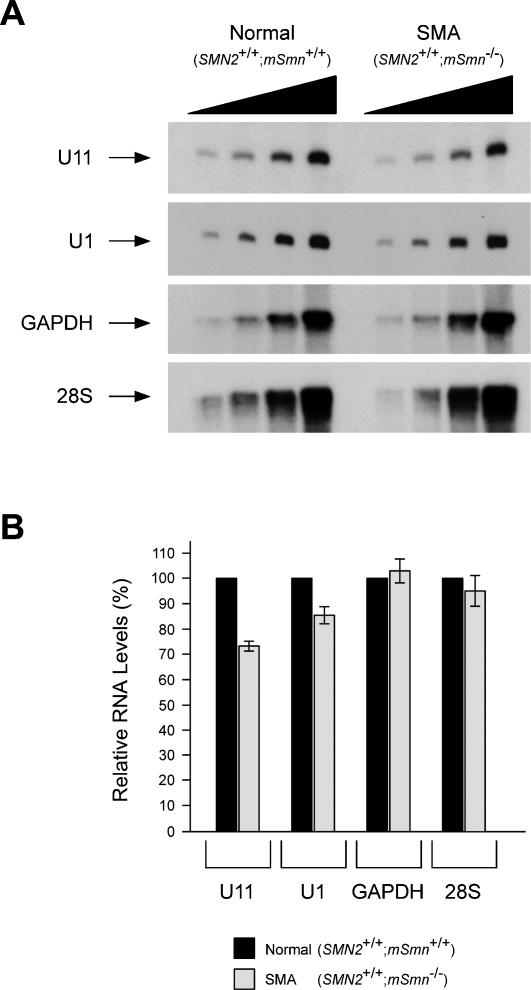
Analysis of endogenous U1 and U11 snRNA levels in the spinal cord of severe SMA mice. (A) Northern blot analysis of four increasing concentrations (0.5, 1, 2 and 4 µg) of total RNA from whole spinal cord extracts of five normal (*SMN2* ^+/+^;*mSmn* ^+/+^) and five severe SMA (*SMN2^+/+^*;*mSmn^−/−^*) mice at postnatal day 3 with probes specific for the RNAs indicated on the left. (B) The intensity of signal of individual RNAs at each concentration was quantified using a STORM 860 Phosphorimager (Molecular Dynamics) and values in the severe SMA mice were expressed as a percentage of the corresponding ones in normal mice, which are set arbitrarily as 100%. The values are presented as mean±SEM. Normal (black bars); severe SMA (light grey bars).

Monitoring snRNP synthesis in mouse tissues with pulse-labeling experiments such as those described above is not feasible. To address the *in vivo* consequence of SMN deficiency on snRNP metabolism in the spinal cord of severe SMA mice, we analyzed the steady-state levels of endogenous snRNPs. To do so, we carried out immunoprecipitation experiments with anti-Sm antibodies from equal amounts of whole spinal cord extracts from normal (*SMN2* ^+/+^;*mSmn* ^+/+^), carrier (*SMN2* ^+/+^;*mSmn* ^+/−^) and severe SMA (*SMN2* ^+/+^;*mSmn* ^−/−^) mice at postnatal day 3. Immunoprecipitated snRNAs were then labeled at the 3′-end with [^32^P] pCp and analyzed by electrophoresis on denaturing gels and autoradiography, thereby allowing simultaneous assessment of Sm core-containing snRNAs. Figure 6 shows that the levels of at least some snRNPs are decreased in spinal cord extracts from severe SMA mice compared with normal controls. Despite a general trend towards reduction, quantification of snRNA levels from four independent experiments highlights a statistically significant decrease (p<0.05) in the levels of U1, U2, U11 and U12 but not of U4 or U5 snRNAs in the spinal cord of severe SMA mice when compared with carrier mice (Figure 7). Remarkably, U11 is the most severely affected with an average 60% reduction (p = 0.018). These findings were further confirmed by Northern blot analysis of U1 and U11 snRNA levels, which are reduced by 15% and 30% in the spinal cord of severe SMA mice, respectively (Figure 8). Northern blot analysis of total snRNA levels does not distinguish between snRNAs that contain the Sm core and those that do not, and the snRNA decrease is smaller than measured with immunoprecipitation experiments. This observation raises the possibility that a significant fraction of snRNAs is not associated with Sm proteins in SMA tissues. Similar analyses were performed using brain and kidney extracts from the same mice. Interestingly, the consequence of low SMN expression in these tissues differs from the spinal cord because only the levels of U11 and U12 snRNPs in brain and of U11 snRNP in kidney are significantly reduced in severe SMA mice compared with carrier mice (Figure 7). Collectively, these results show that the levels of a subset of snRNPs are reduced in tissues from severe SMA mice, and that defective SMN complex activity in snRNP assembly preferentially affects the accumulation of U11 snRNP of the minor splicing pathway *in vivo*. Thus, SMN deficiency unevenly changes the snRNP profile of SMA tissues.

## Discussion

Despite steady progress in our understanding of the genetic basis of SMA, the molecular functions of SMN and the development of animal models, the defect responsible for the specific degeneration of motor neurons in the spinal cord of SMA patients remains elusive. The best-characterized activity of the SMN complex is the assembly of Sm proteins onto snRNAs forming the spliceosomal snRNPs [20]–[23]. These abundant nuclear RNPs function in the context of a dynamic macromolecular machine known as the spliceosome to carry out the excision of introns and ligation of exons to form mature mRNAs [44]. With the goal of understanding SMA pathophysiology, here we have characterized SMN complex activity and the consequence of its deficiency on snRNP metabolism in the spinal cord of mouse models of the disease.

We demonstrated that the levels of a subset of Gemin proteins (namely Gemin2, Gemin6 and Gemin8) are significantly reduced in the spinal cord of severe SMA mice compared with normal mice, and that Gemin8 is an integral component of the SMN complex whose expression is most severely affected by decreased SMN (Figures 1 and S1). In contrast, the levels of Gemin4 and unrip as well as those of several other SMN-interacting proteins are unchanged. The SMN–dependent reduction of Gemin proteins expression has also been observed in cultured cell lines and likely results from degradation due to defective incorporation into SMN complexes [27], [29], [31], [45]. Importantly, the degree of reduction in Gemin2, Gemin6 and Gemin8 expression in the spinal cord of SMA mice correlates with disease severity (Figure 4A), suggesting that these proteins might be involved in the cellular activities of SMN whose impairment underlies SMA pathology. In this regard, it is noteworthy that the beneficial effect of treatment with trichostatin A on survival of SMNΔ7 SMA mice correlates with increased levels of functional SMN complexes in the CNS [46]. Decreased levels of Gemin2, Gemin6 and Gemin8–which are required for efficient snRNP assembly activity [27]–[31]–may also exacerbate the consequence of reduced SMN expression on Sm core formation in the spinal cord of SMA mice. Indeed, we demonstrated that snRNP assembly activity is dramatically reduced in spinal cord extracts from severe SMA mice (Figure 3). A similar decrease in SMN levels and severe impairment of Sm core formation are also observed in extracts from the other SMA tissues analyzed here (Figures 2 and 3). Although quantitative differences exist in the snRNP assembly activity of distinct normal tissues and possibly also in the extent of its decrease in SMA tissues, these results indicate that reduced capacity for Sm core formation is a general biochemical deficiency in severe SMA mice. Importantly, we showed that the degree of snRNP assembly impairment in the spinal cord of SMA mice correlates with disease severity (Figure 4B and 4C). Collectively, our findings provide strong support to the possibility that deficiencies in snRNP biogenesis contribute to SMA pathology.

For the first time, we demonstrated here that the impairment of SMN function in Sm core formation leads to a significant decrease in the levels of a subset of snRNPs in the spinal cord as well as other tissues of severe SMA mice (Figures 6, 7 and 8). As indicated also by similar levels of SmB protein in the spinal cord of normal, carrier and SMA mice (Figure 1), the bulk of snRNPs is however only marginally affected in SMA tissues. This is especially remarkable considering the ten-fold decrease in snRNP assembly activity in neural tissues from SMA mice. It is also in agreement with the observation that steady-state snRNP levels are unchanged in DT40 cells with severely reduced SMN levels and in *Drosophila* larvae harboring SMN null mutations [32], [36]. In light of the striking discrepancy between the degree of reduction in snRNP assembly and snRNP levels in tissues of SMA mice as well as other model systems, it is important to consider that *in vitro* snRNP assembly assays measure the capacity of Sm core formation in extracts, which likely corresponds to the amount of SMN complexes bound to Sm proteins and therefore capable of snRNP assembly. However, reduced snRNP assembly capacity *in vitro* as a consequence of low SMN levels may not necessarily translate into a defect of snRNP synthesis *in vivo* if the SMN complexes are in excess. In order for snRNP accumulation to be affected *in vivo*, the SMN complex must fail to meet the demand for Sm core formation on the snRNAs, which is dictated primarily by the rate of snRNA transcription. Thus, the physiologically critical parameter is the threshold of SMN activity required to support the level of snRNP synthesis needed *in vivo*, which likely differs in distinct cells and changes also with development. Only if SMN activity decreases below this threshold insufficient snRNPs are made and steady-state snRNP levels may be affected. Although it is generally assumed that snRNP assembly is rate limiting in SMA, we found surprisingly that snRNP synthesis *in vivo* is not affected in type I SMA fibroblasts (Figure 5). Together with the overall modest reduction of snRNP levels in SMA tissues, these results suggest that SMN complexes competent for snRNP assembly greatly exceed the amount needed to meet the *in vivo* demand for snRNP synthesis in most tissues and that decreased SMN levels have little if any impact on snRNP accumulation in SMA. An important implication is that, somewhat similar to the situation in larval lethal Drosophila mutants [36], the specific neuromuscular phenotype triggered by systemic SMN deficiency in mouse models of SMA is not caused by global depletion of spliceosomal snRNPs. Nevertheless, our finding that the levels of a subset of snRNPs are significantly decreased in tissues of severe SMA mice indicates that there are at least some cells in SMA in which SMN capacity for snRNP assembly becomes overwhelmed and insufficient snRNPs are made possibly due to a requirement for high levels of snRNP synthesis.

One of the most strikingly unexpected findings of our study is that impairment of SMN ubiquitous function specifically alters the snRNP profile of SMA tissues by decreasing the levels of a subset of snRNPs and more prominently of U11. Spliceosomal snRNPs are divided into two distinct classes depending on the type of introns that they remove [47]. The vast majority of eukaryotic introns are processed by the major (or U2-dependent) spliceosome formed by U1, U2, U4/U6 and U5 snRNPs. Less than 1% of introns are processed by the minor (or U12-dependent) spliceosome comprising U11, U12, U4atac/U6atac and U5 snRNPs. Specific conserved sequence features distinguish the two types of introns and determine their commitment to either the major or the minor pre-mRNA splicing pathway [47], [48]. Bioinformatic analysis of intron sequences from the databases reveals the presence of approximately seven hundred U12-type introns in the human genome [49]–[51]. Consistent with the relatively low abundance of the target introns, minor snRNAs (10^3^–10^4^ per cell) are two orders of magnitude less abundant than major snRNAs (10^5^–10^6^ per cell) [52]. Although the SMN complex assembles the Sm core on both major and minor snRNAs [26], [53], differences in the abundance, efficiency of Sm core formation and turnover rate of individual snRNAs might contribute to the observed effects of SMN deficiency on the levels of specific snRNPs. Future studies will be needed to address these issues directly. Regardless of the mechanism(s), however, our results indicate that SMN deficiency unevenly alters the physiological proportion of endogenous snRNPs in tissues of severe SMA mice, and preferentially affects the accumulation of U11 snRNP of the minor splicing pathway. These findings have important potential implications for SMA pathogenesis because the disease trigger targeted by SMN reduction may lie within genes containing introns processed by the minor splicing pathway. There is evidence that processing of U12-type introns is slower and possibly more error prone than that of U2-type introns under normal conditions and might represent a rate-limiting step in the expression of genes that contain these introns [50], [54]. Decreased levels of minor snRNPs such as U11 may further enhance this situation and have deleterious consequences on the expression of mRNAs containing U12-type introns in cells of SMA patients. It is also conceivable that although the minor splicing pathway processes a number of genes only a few may be affected by SMN reduction because of differences in the splicing efficiency of individual U12-type introns.

Dysfunctions in components of both constitutive and regulated pre-mRNA splicing have been implicated in the disease mechanisms of an increasing number of human disorders [55], [56]. In addition, there is precedent for a model where reduction of a particular splicing factor results in a tissue-specific phenotype. Myotonic dystrophy is caused by nucleotide repeat expansions in the non-coding region of specific mRNAs, which act to sequester splicing factors such as the muscle blind protein away from the pre-mRNA processing machinery leading to aberrant processing of distinct target mRNAs in different tissues [57]. As a consequence of this splicing deregulation, in mouse models of myotonic dystrophy, the chloride channel 1 gene is processed incorrectly during early postnatal development of skeletal muscle and the fetal mRNA form that is not active in chloride conductance is retained [58]. In turn, this affects the physiological increase in chloride channel conductance in skeletal muscle resulting in myotonia and muscle degeneration [59]. It is tantalizing to speculate that the processing of one or more introns in specific genes that have a critical role in motor neuron biology might be similarly affected by reduced functionality of the U12-dependent spliceosome in SMA. It is noteworthy that U12-dependent introns are not randomly distributed in the genome but rather enriched in few gene families [48]–[51]. Members of the voltage-gated ion channel gene family contain an unusually high frequency of U12-dependent introns and are especially interesting in the context of SMA pathology [48]. These genes control numerous activities that are critical for neuronal function and muscle contraction, including action potential, signaling processes and synaptic transmission, and whose deficiency is responsible for several neuromuscular and neurological human disorders [60]–[62]. One attractive albeit presently speculative scenario is that altered processing of some members of this gene family could cause electrophysiological disturbances that contribute to motor unit dysfunction and SMA pathophysiology. It should be pointed out however that there is no evidence of splicing defects in SMA and it is not known whether the reduction of snRNP levels reported here would indeed affect the functionality of the minor pre-mRNA splicing pathway. Future studies will be needed to address the many questions stemming from our work. In particular, whether the minor splicing pathway rather than the major splicing pathway is affected in SMA and whether defective processing of specific mRNAs containing U12-dependent introns contributes to the preferential vulnerability of motor neurons to reduced levels of SMN.

## Materials and Methods

### Animals and tissue harvesting

Severe SMA mice were produced from carrier parents of the genotype *SMN2^+/+^;mSmn^+/−^* (line 89; *FVB.Cg-Tg(SMN2)89Ahmb Smn1^tm1Msd^*) [16]. SMNΔ7 SMA mice were generated from males and females of the genotype *SMN2^+/+^;SmnΔ7^+/+^;mSmn^+/−^* (line 4299; FVB.Cg-Tg(SMN2*delta7)4299Ahmb Tg(SMN2)89Ahmb Smn1^tm1Msd^) [18]. SMN(A2G) SMA mice were bred from males with the genotype *SMN2^+/+^;SMN(A2G)^+/+^;mSmn^+/−^* (line 2023; FVB.Cg-Tg(SMN1*A2G)2023Ahmb Tg(SMN2)89Ahmb Smn1^tm1Msd^) and females with the genotype *SMN2^+/+^;mSmn^+/−^* [19]. High copy SMN2 SMA mice were produced by mating males of the genotype *SMN2(89)^+/+^;SMN2(566)^+/+^;mSmn^+/−^* with females of the genotype *mSmn^+/−^* [16]. All experiments were conducted in accordance with the protocols described in the National Institutes of Health *Guide for the Care and Use of Animals* and were approved by the Ohio State University Institutional Laboratory Animal Care and Use Committee. Mouse tissues were harvested from neonatal pups at postnatal day 3 (with birth being defined as postnatal day 1). The pups were lightly anesthetized with isoflurane and were then euthanized by rapid decapitation. Forebrains, spinal cords and kidneys were rapidly dissected and immediately frozen in liquid nitrogen. All tissue samples were stored at −80°C until use. A tail biopsy, which was used for genotyping, was also taken from each pup.

### Genotyping

Neonatal offspring were genotyped using a PCR-based assay on genomic DNA from tail biopsies—obtained after death—as described previously [16]–[19], [63]. The presence of the *mSmn* knockout allele was determined by PCR amplification of NeoR/mSmn junction site: NeoB, 5′-gcagctgtgctcgacgttgtc-3′ and SmnInt2R, 5′-taagaaagcctcgacgttgtc-3′ (PCR conditions: 95°C for 4 min, 35 cycles of 95°C for 1 min, 63°C for 1.5 min and 72°C for 1 min followed by a final extension at 72°C for 4 min). PCR primers designed to detect an intact *mSmn* allele were used to distinguish SMA pups from carrier pups: mSmnEx2AF, 5′-ttttctccctcttcagagtgat-3′ and mSmnEx2BR, 5′-ctgtttcaagggagttgtggc-3′ (PCR conditions: 95°C for 4 min, 32 cycles of 95°C for 1 min, 57°C for 1 min and 72°C for 1 min followed by a final extension at 72°C for 4 min). SMA mice (*mSmn^−/−^*) would be positive for the mSmn knockout PCR reaction and negative for the intact mSmn PCR reaction while carrier mice (*mSmn^+/−^*) would be positive for both the mSmn knockout and intact mSmn PCR reactions. Normal mice (*mSmn^+/+^*) would test negative for the mSmn knockout PCR but positive for the intact mSmn PCR reaction. Both the SMN(A2G) and SMNΔ7 transgenes were identified by using the following PCR primers: SMNex4.5F, 5′-actgggaccaggaaagccaggt-3′ and SMNex6R, 5′-gccagtatgatagccactcatg-3′ (PCR conditions: 95°C for 4 min, 32 cycles of 95°C for 1 min, 57°C for 1 min and 72°C for 1 min followed by a final extension at 72°C for 4 min). The *SMN2* transgene (both lines 89 and 566) was detected by the following PCR reaction: SMN2F2, 5′-gcgatagagtgagactccatct-3′ and SMN2R1, 5′-gacatagaggtctgatctttagct-3′ (PCR conditions: 95°C for 4 min, 32 cycles of 95°C for 1 min, 57°C for 1 min and 72°C for 1 min followed by a final extension at 72°C for 4 min). To distinguish *SMN2* (line 89) from *SMN2* (line 566), we performed the above PCR using a limited number of 20 cycles and normalized band intensity to that obtained from PCR-based detection of the endogenous, single-copy gene, IL-2 (IL2-F, 5′-ctaggccacagaattgaaagatct-3′ and IL2-R, 5′-gtaggtggaaattctagcatcatcc-3′) [64].

### Antibodies

The antibodies used in this study were as follows: anti-SMN clone 8 (BD Transduction Laboratories), anti-Gemin2 2E17 [65], anti-Gemin4 17D10 [66], anti-Gemin6 20H8 [28], anti-Gemin8 1F8 [27], anti-unrip 3G6 [27], anti-Sm Y12 (Lab Vision), anti-hnRNP R/Qs 18E4 [67], anti-H1 and core histones (Chemicon), anti-tubulin DM 1A (Sigma), anti-Profilin I and anti-Profilin II (kindly provided by Dr. Witke).

### Cell culture and treatments

Human fibroblast cell lines from one normal carrier individual (GM03814) and four type I SMA patients (GM00232, GM09677, GM03813, GM03815) were grown in RPMI 1640 medium (BioWhittaker) containing 15% fetal bovine serum, 2 mM glutamine and 1% penicillin/streptomycin. In pulse-labeling experiments to monitor snRNP synthesis *in vivo*, subconfluent human fibroblasts were incubated for 6 h with 100 µCi of [^32^P] phosphoric acid in medium without phosphate.

### Extract preparation

Total protein extracts for Western blot analyses were prepared by homogenization of frozen tissues in SDS/PAGE sample buffer followed by brief sonication and boiling. Whole cell and tissue extracts were prepared by homogenization of frozen tissues with ice-cold reconstitution buffer (20 mM Hepes-KOH pH 7.9, 50 mM KCl, 5 mM MgCl_2_, 0.2 mM EDTA, 5% glycerol) containing 0.01% NP40 as previously described [42]. Homogenates were then passed five times through a 25G needle and centrifuged 15 min at 10,000 rpm at 4°C. Supernatants were collected and protein concentration measured using the Lowry method (Biorad). Extracts were then used directly for snRNP assembly experiments or stored in frozen aliquots at −80°C for Western blot and immunoprecipitation experiments. All protein analyses were carried out by SDS/PAGE on 12% polyacrylamide gels and Western blot.

### *In vitro* assembly of snRNPs

U1 snRNA was transcribed in vitro from a linearized template DNA in the presence of [α^32^P] UTP (3000 Ci/mmol) and m7G cap analogue (New England Biolabs), and then purified from denaturing polyacrylamide gels according to standard procedures. The snRNP assembly reactions were carried out for 1 h at 30°C in a volume of 20 µl of reconstitution buffer containing 0.01% NP40, 25 µg of whole tissue extracts, 10,000 cpm of in vitro transcribed [α^32^P] UTP-labeled U1 snRNA, 2.5 mM ATP and 10 µM E.coli tRNA [26], [42]. Reactions were then analyzed by immunoprecipitation with anti-Sm (Y12) antibodies or, following addition of heparin and urea to a final concentration of 5 mg/ml and 2 M, respectively, by electrophoresis on 6% polyacrylamide native gels at 4°C and autoradiography.

### Immunoprecipitation experiments

Immunoprecipitations with anti-Sm (Y12) antibodies from snRNP assembly reactions or whole tissue extracts (200 µg) were carried out in RSB-500 buffer (500 mM NaCl, 10 mM Tris-HCl pH 7.4, 2.5 mM MgCl_2_) containing 0.1% NP40, EDTA-free protease inhibitor cocktail (Roche) and phosphatase inhibitors (50 mM NaF, 0.2 mM Na_3_VO_4_) for 2 h at 4°C. After five washes with the same buffer, bound RNAs were recovered from immunoprecipitates by proteinase K treatment, phenol/chloroform extraction and ethanol precipitation. 3′-end labeling experiments were carried out in the presence of [^32^P] pCp (3000Ci/mmol) and T4 RNA ligase (Roche) following manufacturer's instructions, and unincorporated nucleotides were removed by centrifugation through micro Bio-Spin P-30 columns (Biorad). RNAs were analyzed by electrophoresis on 6% polyacrylamide/8M urea gels and autoradiography. Immunoprecipitated snRNAs were quantified using a STORM 860 Phosphorimager (Molecular Dynamics) and the ImageQuant version 4.2 software. Two-tailed P values were calculated using the unpaired Student t test and the InStat software.

### Northern blot analysis

Total RNA from whole spinal cord extracts was analyzed by electrophoresis on 6% polyacrylamide/8M urea gel or 1% agarose-formaldehyde gel and transferred to a nylon membrane (Genescreen Plus, Perkin Elmer). Antisense RNA probes corresponding to mouse U1 (from nucleotide 122 to 157), mouse U11 (from nucleotide 58 to 82), mouse 28S ribosomal RNA (from nucleotide 4400 to 4515) and mouse GAPDH mRNA (from nucleotide 345 to 660) were transcribed *in vitro* in the presence of [α^32^P] UTP (3000 Ci/mmol) using the MEGAshortscript T7 kit (Ambion). RNA probes (0,75×10^6^ cpm/ml) and sheared salmon sperm DNA (200 µg/ml) were denatured at 100°C for 5 min and then transferred to ice. Hybridization was carried out overnight at 50°C in hybridization buffer (1 M NaCl, 10% dextran sulphate, 50% formamide, 1% SDS).

## Supporting Information

Figure S1 SMN knockdown affects the levels of a subset of Gemin proteins in HeLa cells. (A) HeLa S3 cells were transfected with siRNAs against SMN, Gemin8 and luciferase (control) that have been previously described [27]–[29]. 72 hours post-transfection, equal amounts of total proteins were analyzed by Western blot with antibodies against the proteins indicated on the left. SMN knockdown in HeLa cells markedly affects the levels of Gemin2, Gemin6, Gemin7 and Gemin8 but not of Gemin3, Gemin4, Gemin5 and unrip. Note that Gemin2 decrease is not observed upon Gemin8 knockdown and thus specific for SMN reduction. (B) To quantify the decrease of Gemin proteins that are affected by SMN knockdown, total proteins from HeLa cells in which Gemin8 and SMN levels were reduced by RNA interference as in (A) were analyzed together with the indicated serial dilutions of total proteins from HeLa cells treated with siRNAs against luciferase (control). Proteins were analyzed by Western blot with antibodies against the proteins indicated on the left. For each Gemin protein, direct comparison of signal intensity detected by western blot in the SMN RNAi sample and in serial dilutions of the control RNAi sample indicates that SMN decrease most prominently affects the levels of Gemin8 followed by Gemin2, Gemin6 and Gemin7.
(0.90 MB TIF)

Figure S2 In vitro snRNP assembly activity in kidney of severe SMA mice. Equal amounts of whole tissue extracts (25 μg) from the kidney of normal (SMN2+/+;mSmn+/+), carrier (SMN2+/+; mSmn+/−) and severe SMA (SMN2+/+;mSmn−/−) mice at postnatal day 3 were analyzed in snRNP assembly reactions with radioactive U1 snRNA followed by immunoprecipitation with anti-Sm (Y12) antibodies. Input (2.5%) and immunoprecipitated U1 snRNAs were analyzed by electrophoresis on denaturing polyacrylamide gels and autoradiography.
(0.14 MB TIF)
