# Supplementary figures and images for: Ribonucleoprotein Assembly Defects Correlate with Spinal Muscular Atrophy Severity and Preferentially Affect a Subset of Spliceosomal snRNPs
Source: PLoS One. 2007 Sep 26;2(9):e921. doi: 10.1371/journal.pone.0000921 (PMC1976558; doi:10.1371/journal.pone.0000921)

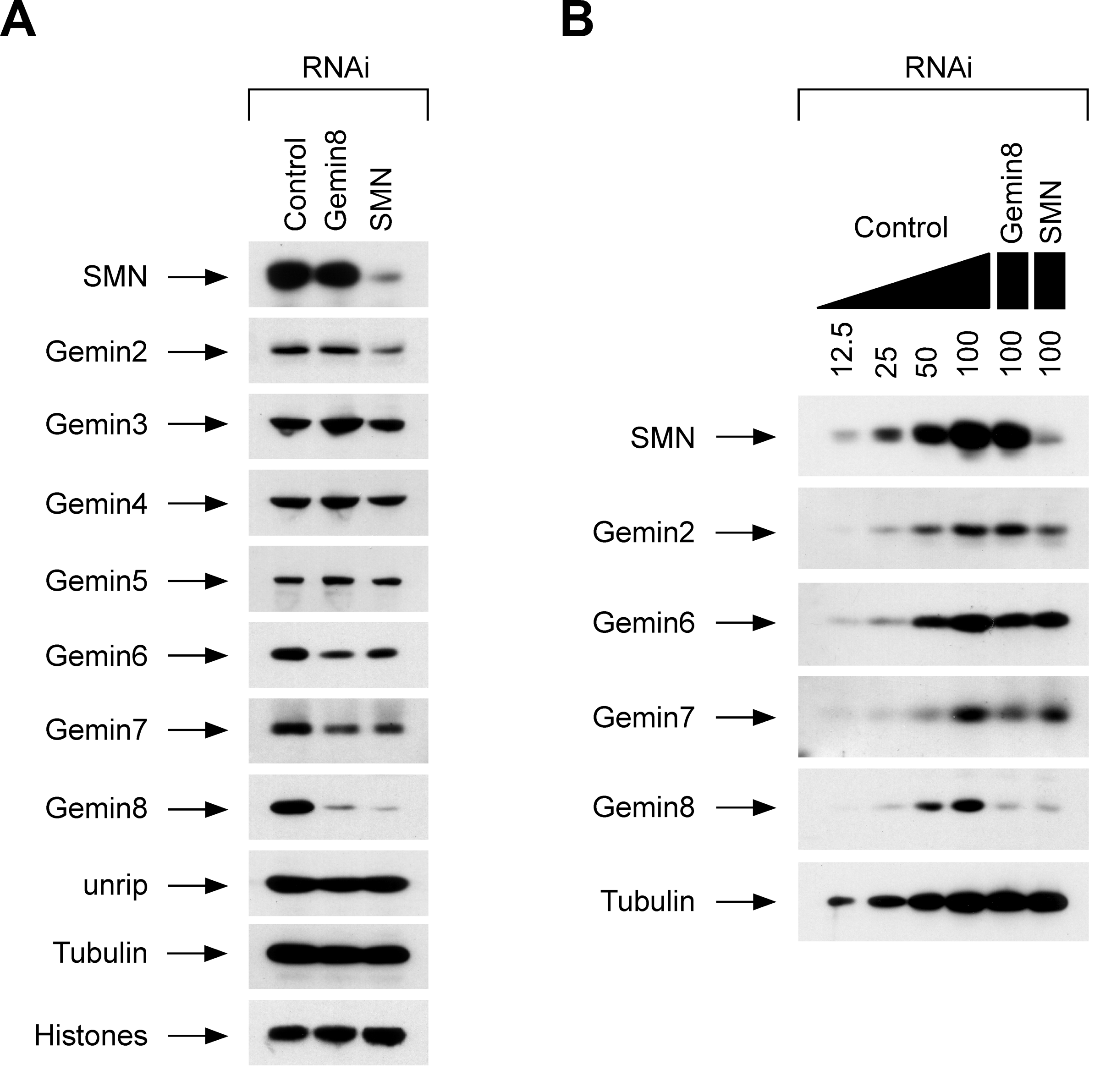

Supplement: Figure S1 — SMN knockdown affects the levels of a subset of Gemin proteins in HeLa cells. (A) HeLa S3 cells were transfected with siRNAs against SMN, Gemin8 and luciferase (control) that have been previously described [27]–[29]. 72 hours post-transfection, equal amounts of total proteins were analyzed by Western blot with antibodies against the proteins indicated on the left. SMN knockdown in HeLa cells markedly affects the levels of Gemin2, Gemin6, Gemin7 and Gemin8 but not of Gemin3, Gemin4, Gemin5 and unrip. Note that Gemin2 decrease is not observed upon Gemin8 knockdown and thus specific for SMN reduction. (B) To quantify the decrease of Gemin proteins that are affected by SMN knockdown, total proteins from HeLa cells in which Gemin8 and SMN levels were reduced by RNA interference as in (A) were analyzed together with the indicated serial dilutions of total proteins from HeLa cells treated with siRNAs against luciferase (control). Proteins were analyzed by Western blot with antibodies against the proteins indicated on the left. For each Gemin protein, direct comparison of signal intensity detected by western blot in the SMN RNAi sample and in serial dilutions of the control RNAi sample indicates that SMN decrease most prominently affects the levels of Gemin8 followed by Gemin2, Gemin6 and Gemin7. (0.90 MB TIF) [file pone.0000921.s001.tif]

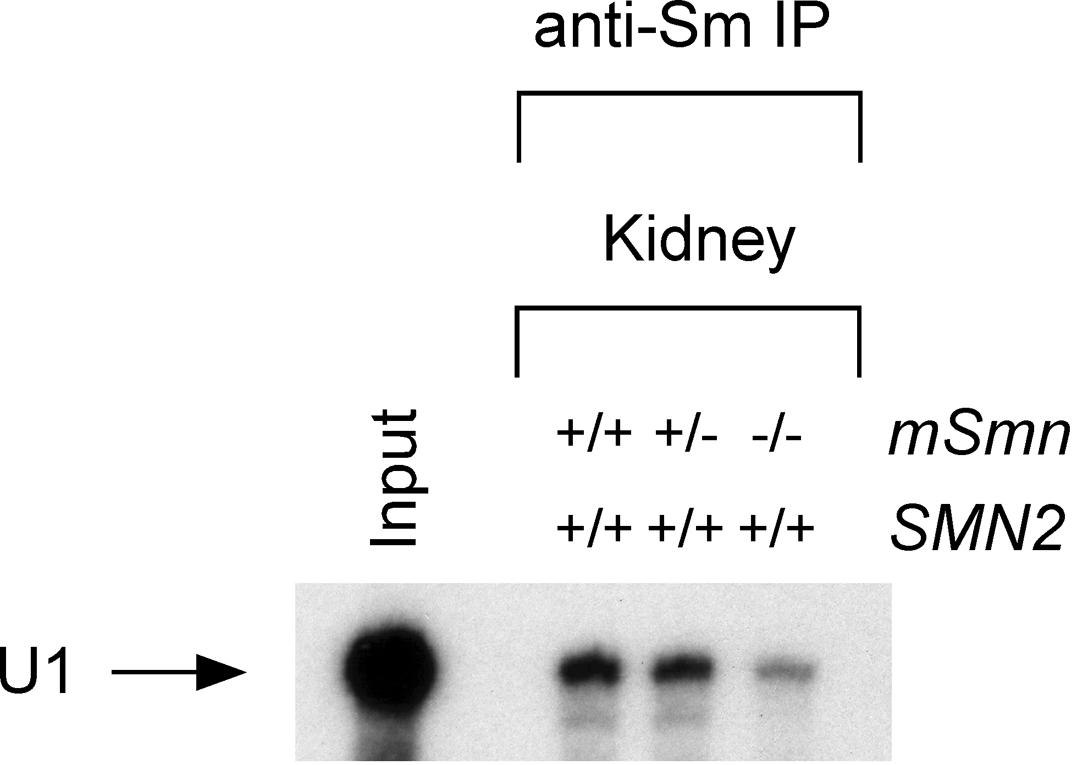

Supplement: Figure S2 — In vitro snRNP assembly activity in kidney of severe SMA mice. Equal amounts of whole tissue extracts (25 μg) from the kidney of normal (SMN2+/+;mSmn+/+), carrier (SMN2+/+; mSmn+/−) and severe SMA (SMN2+/+;mSmn−/−) mice at postnatal day 3 were analyzed in snRNP assembly reactions with radioactive U1 snRNA followed by immunoprecipitation with anti-Sm (Y12) antibodies. Input (2.5%) and immunoprecipitated U1 snRNAs were analyzed by electrophoresis on denaturing polyacrylamide gels and autoradiography. (0.14 MB TIF) [file pone.0000921.s002.tif]
